# Feasibility of prototype diamond detectors for pulsed UHDR PBS small-field proton dosimetry for proton FLASH experiments

**DOI:** 10.1088/1361-6560/ae023b

**Published:** 2025-09-22

**Authors:** Jufri Setianegara, Aoxiang Wang, Nicolas Gerard, Jarrick Nys, Mark Szczepanski, Hao Gao, Yuting Lin

**Affiliations:** 1 Department of Radiation Oncology, University of Pennsylvania, Philadelphia, PA, United States of America; 2 Department of Radiation Oncology, University of Kansas Medical Center, Kansas City, KS, United States of America; 3 Department of Biomedical Engineering, Huazhong University of Science and Technology, Wuhan, Hubei, People’s Republic of China; 4 Ion Beam Applications (IBA), Louvain-la-Neuve, Wallonia, Belgium; 5 Physikalisch-Technische Werkstätten (PTW), Freiburg, Baden-Württemberg, Germany; 6 University of Texas Southwestern Medical Center, Dallas, TX, United States of America

**Keywords:** pulsed proton UHDR, PBD UHDR protons, diode detectors, diamond detectors, UHDR charge saturation, small-field UHDR protons

## Abstract

*Objective.* This study aims to investigate the responses of prototype diamond detectors under pulsed ultra-high dose rates (UHDRs) pencil-beam-scanning (PBS) protons from a compact proton synchrocyclotron (IBA Proteus®ONE) for small-field UHDR dosimetry. *Approach*. flashDiamond detectors (fDs) were cross-calibrated with their relative proton responses characterized at conventional dose rates (CONV). Then, absolute UHDR dosimetry was performed and small-field response assessed. These experiments were also conducted with Razor Diode and microdiamond detectors (mDs) for cross-reference. Cross-calibrations were performed against an ADCL-calibrated PPC05 plane-parallel ionization chamber with 59.23 cGy nC^−1^ calibration coefficients. fD’s linearity, dose-rate, energy, and linear-energy-transfer (LET) responses were assessed under CONV protons. Pulsed UHDR PBS protons of 228 MeV were produced from a medical proton synchrocyclotron (IBA Proteus®ONE) for 1.5 × 1.5–3.0 × 3.0 cm^2^ square fields. Nominal absolute UHDR dosimetry was performed at 3 × 3 cm^2^ field sizes with relative responses at smaller fields benchmarked against it. *Main results*. fD had 28.6 ± 0.1 cGy nC^−1^ sensitivities under CONV protons and were linear in response with dose-rate independence within ±0.50%. fD were similar to mD in proton energy and LET responses. However, there is an over-response of approximately 5.49%, 6.51% and 13.7% at the 226, 150 and 70 MeV Bragg peaks respectively. Under pulsed proton UHDR irradiation (0.80% s.t.d, 32.6 ± 0.5 cGy dose-per-pulse), fD responded within ±1% as PPC05 with negligible saturation. fD agreed within ±1% with other comparable small-field detectors under small-field UHDR beams and within ±2% of RayStation treatment planning system calculations. There is negligible partial volume averaging with fDs. *Significance*. Novel fD detectors did not saturate under pulsed UHDR PBS proton irradiation. Their miniscule active crystals make them suitable for small-field dosimetry but render them relatively insensitive compared to mDs. When cross-calibrated, they are suitable for absolute small-field UHDR dosimetry or for relative exit dosimetry monitoring purposes during UHDR radiobiological experiments.

## 1. Introduction

FLASH radiotherapy refers to the delivery of therapeutic radiation doses at an ultrahigh dose rate (UHDR) and it has been shown to elicit differential biological responses between normal cells and cancer cells (Esplen *et al* 2020, Lin *et al* 2021a, Matuszak *et al* 2022, Gao *et al* 2022b, Limoli and Vozenin 2023, Tang *et al* 2024). This differential response is referred to in literature as the ‘FLASH effect’ and has been demonstrated in early preclinical experiments (Kacem *et al* 2022, Lin *et al* 2022, McGarrigle *et al* 2024, Ma *et al* 2024b). While it is well-established that the FLASH effect is generally induced at UHDRs above 40 Gy s^−1^ and above dose thresholds of approximately 10 Gy (van Marlen *et al* 2022, Fenwick *et al* 2024), there has been recent radiobiological investigations to obtain a quantifiable and mechanistic understanding of the underlying radiobiology leading to its induction (Hageman *et al* 2022, Lin *et al* 2022, Chow and Ruda 2024, Yan *et al* 2024, Ma *et al* 2024b). In addition to FLASH radiobiology, FLASH research has also rapidly evolved to include clinical trials (Taylor *et al* 2022, Mascia *et al* 2023, Daugherty *et al* 2024) along with the developments of various associated FLASH-specific treatment planning methods to optimize dose coverage and the FLASH effect (Gao *et al* 2020, 2022a, Wei *et al* 2021, Lin *et al* 2021b, Kang *et al* 2022, Liu *et al* 2023, Ma *et al* 2024a).

Currently, UHDR irradiations have been achieved for all clinically utilized radiation modalities namely photons (Montay‐Gruel *et al* 2022), electrons (Giannini *et al* 2024) and protons (Hughes and Parsons 2020, Diffenderfer *et al* 2022, Atkinson *et al* 2023). These UHDR irradiations have been delivered on a wide variety of machines which include medical linear accelerators and cyclotrons that have been modified for UHDR deliveries as well as research beamlines and non-medical accelerators (Patriarca *et al* 2018, Lyu *et al* 2021, Rahman *et al* 2021, Kroll *et al* 2022, Gao *et al* 2022b, Tan *et al* 2023). Despite the wide variety of UHDR capable accelerators, its clinical translation (Vozenin *et al* 2022) has been delayed by the lack of reproducibility of preclinical UHDR experiments between different investigators despite supposed similarities in the reported UHDR experimental conditions (Kim *et al* 2021, Zhang *et al* 2023). As such, there has been recent efforts pushing for a more complete reporting of the UHDR temporal beam structures such as instantaneous dose rates, pulse width and dose per pulse (DPP) which can vary significantly across multiple institutions despite having similar reported dose rates (Schuler *et al* 2022). This is further supported by recent preclinical evidence demonstrating the dependence of the FLASH effect induction and magnitudes on the pulse structure of the radiation beam, namely dose rates and DPP, in zebrafish embryos and C57BL/6J mice (Karsch *et al* 2022, Bell *et al* 2025). Of note, there is a better sparing of the regenerating crypt assays and a reduction of gastrointestinal toxicities of C57BL/6J mice when irradiated with UHDR beams with a higher DPP.

Radiobiological experiments utilizing UHDR proton beams are increasingly performed due to the dosimetric advantages of protons in inducing the FLASH effect more effectively at deeper depths and the growing availability of UHDR-capable medical proton accelerators (Darafsheh *et al* 2020, Diffenderfer *et al* 2020, Atkinson *et al* 2023, Lourenco *et al* 2023, Yang *et al* 2023, Yin *et al* 2024, Zeng *et al* 2024). The bulk of preclinical proton UHDR irradiations were performed with isochronous medical cyclotrons with a quasi-continuous pulse structure (Bell *et al* 2025, McManus *et al* 2020). While these cyclotrons can achieve maximum nozzle currents in the hundreds of nAs and thus, deliver dose rates that are multiple times that of the dose rate thresholds to induce the FLASH effect (Jolly *et al* 2020, Spruijt *et al* 2024), their quasi-continuous pulse structure (Darafsheh *et al* 2020) will result in proton UHDR beams of relatively low DPP. In contrast, synchrocyclotrons have a unique pulse structure (Darafsheh *et al* 2020) and can attain even greater instantaneous proton currents in the order of *μ*A (Darafsheh *et al* 2021) which can result in proton UHDR beams with a higher DPP and allowing for interesting radiobiological experiments investigating the dependence of proton-induced FLASH effects on the dose rates and DPP (Karsch *et al* 2022, Deffet *et al* 2023, Sorensen *et al* 2024). However, the accurate measurement of radiation doses from these UHDR proton beams with high DPP values can be experimentally challenging as compared to their quasi-continuous UHDR counterparts. For ionization chambers, high DPP values will result in high rates of initial recombinations and thus result in high ion recombination factors as seen previously for ionization chambers that were exposed to very high electron energy UHDR beams (McManus *et al* 2020). Semiconductor detectors such as diamond detectors and diodes can be used to complement ionization chamber readings. While they generally do not have ADCL-traceability, they can be cross-calibrated at conventional proton dose rates against an ionization chamber with an ADCL-traceable calibration factor and thereafter, be used for proton UHDR dosimetry. Unlike ionization chambers, these semiconductor detectors do not have any recombination effects. In addition, these detectors generally have a lower work function and hence, can potentially result in higher signal-to-noise ratios (SNRs) as compared to ionization chambers. These higher SNRs allow for these dosimeters to be miniaturized and used for small field dosimetry which can be potentially useful for preclinical proton UHDR experiments.

However, UHDR beams with high DPPs or instantaneous dose rates can result in charge saturation effects within the solid-state semiconductor detectors that can manifest as non-linear measurement artifacts. Previously, irreversible charge saturations were noticed for microdiamond detectors (mDs) when exposed to electron UHDR beams beyond a DPP of 15.0 cGy (Di Martino *et al* 2020, Kranzer *et al* 2022). The operation of mD was previously electronically represented as a Schottky diode within an equivalent circuit diagram where potential mD’s imperfections, such as its finite resistivity, conductivity and capacitance, were represented as a series of equivalent capacitors and resistors that are connected either in series or in parallel to a Schottky diode (representing an ideal mD) (Kranzer *et al* 2022). Charge saturation under UHDR beams with a high DPP were modeled by the build-up of generated charges that would accumulate within the mD’s finite capacitance. The finite resistivity of mD will in turn delay the discharge of these accumulated charges which resulted in a significant voltage buildup within the mD’s structure, counteracting the intrinsic potential of its depletion region. This will cause a non-linear detector response manifesting as charge saturation effects. Prototype diamond detectors that are more suited for UHDR will ideally have a lower internal resistance and a lowered sensitivity to prevent the build-up of voltages across the diamond detector at high DPP UHDR beams thus mitigating the occurrence of these saturation effects.

In this work, we characterized the performance of such a prototype flashDiamond detector (fD) under proton irradiations and investigated its suitability to be used for routine proton UHDR dosimetry purposes. fD was first comprehensively characterized (linearity, dose rate dependence, lateral volume averaging, energy dependence, linear-energy-transfer (LET) dependence, small-field dependence) at conventional proton dose rates (CONV). Then, cross-calibrations were performed at CONV protons relative to an ADCL-calibrated PPC05 plane-parallel ionization chamber (PPIC). Finally, the absolute proton UHDR doses from a magnetically scanned pulsed UHDR beam with a high DPP was determined and cross-compared with ion chambers and other similar semiconductor detectors (mD and Razor Diode).

## 2. Methods and materials

### 2.1. IBA Proteus®ONE UHDR beam configuration

The IBA Proteus®ONE at the University of Kansas Proton Center is a compact, single-gantry proton unit. Pulsed proton beams (1 kHz pulse repetition rate, 10 *μ*s pulse duration, 230 MeV nominal energy) are generated from an S2C2 synchrocyclotron which can achieve maximum conventional proton charges of 4.5 pC per pulse at the isocenter (Tan *et al* 2023). Proton energies ranging from 230 MeV to 70 MeV are generated using a wedged variable energy degrader that is located at the cyclotron’s exit. The energy selection system (ESS) of the Proteus®ONE includes beam steering and focusing elements such as dipole and quadrupole magnets and divergence-limiting slits, which are all integrated within the gantry of the Proteus®ONE unit.

The IBA Proteus®ONE’s compact gantry rotates from 325° to 188° and covers a total range of 223°. Proton beams are delivered through a dedicated scanning beam nozzle. The treatment couch rotates from 0° to 180°. Beam positions are controlled by two orthogonal scanning magnets which operate in the *x*- (left-to-right) and *y*-directions (superior-to-inferior). The focal lengths of the proton beams were experimentally measured to be 294.5 cm and 910.7 cm for the *x*- and *y*-directions respectively during beam commissioning. As previously mentioned, continuously-variable proton beam energies (70–226 MeV) are clinically available, with in-air spot sizes (*σ*) continuously varying from 0.344 cm at 226 MeV to 0.771 cm at 70 MeV. Any beam-modifying accessories such as range shifters can be efficiently mounted upon an accessory drawer that is located at the end of the beam nozzle (Tang *et al* 2024). This accessory drawer can extend and retract along the beam’s direction of travel, with maximum and minimum extensions of 45.4 cm and 17.0 cm from the gantry isocenter respectively. In addition to range shifters, the accessory drawer allows for the reproducible and stable mounting of patient-specific or custom-built collimators which allows for novel experimental proton research such as proton minibeam spatially-fractionated radiotherapy (Lin *et al* 2024a, 2024b).

The S2C2 synchrocyclotron achieves UHDRs by increasing the charge per pulse while retaining fixed pulse widths. This is performed by increasing the Dee voltage of the synchrocyclotron’s radiofrequency system, increasing the arc current across the ion source, and tuning the ESS for maximum beam transmission at the maximum proton energy from the synchrocyclotron. Within our clinic, this was tuned at a gantry angle of 0° and hence, all subsequent UHDR irradiations were performed at this gantry angle. During UHDR deliveries, the two beam monitoring ionization chambers (BMICs) within the proton’s nozzle (IC_1_ and IC_2_) were disabled as their associated readout electrometers had a higher tendency to saturate under UHDR protons which will result in higher measurement uncertainties. Instead, beam monitoring of the charge was performed with the cyclotron’s BMIC (IC_cyclo_). Specifically, beam monitoring was performed using an open-loop system. A Faraday cup that was built in-house with a charge collection efficiency of 95.3% was used to establish a correlation between the charge readings between IC_1,2_ and the Faraday cup’s readings under 226 MeV conventional proton beams. This correlation was then used to determine the relationship between clinical proton monitor units (MUs) with the Faraday cup’s readings. Under UHDR irradiations with IC_1,2_ disabled, an initial correlation between the UHDR IC_cyclo_ readings and the Faraday cup’s charge readings was obtained. This relationship was subsequently used to define a ‘FLASH MU’ based on the IC_cyclo_ charge readings that closely match the Faraday cup’s readings that correspond to 1 clinical MU under conventional proton dose rates. The IC_cyclo_ of the S2C2 synchrocyclotron does not have any in-built temperature or pressure sensors and thus daily pressure and temperature corrections are performed at the start of each day by delivering a known FLASH MU amount to the Faraday cup and comparing it with previous values. During each UHDR beam delivery, an initial look-up-table (LUT) is generated which correlates the Dee voltage of the S2C2 to the MU per pulse. The LUT will be used to determine the Dee voltage settings that will result in the MU per pulse, i.e. dose rates, that is requested for the subsequent actual UHDR irradiations. Ion recombination (Zou *et al* 2021, Vidal *et al* 2024, Yin *et al* 2024) for IC_cyclo_ during UHDR deliveries were corrected for by using a correction map that was obtained by delivering similar MU values to the Faraday cup at different dose rates (MU per pulse) and reading the charges that were measured by IC_cyclo_. These correction factors range from 1.000 at the lowest dose rates to 1.017 at the highest dose rates, and these factors will be pre-determined based on the MU per pulse settings of the UHDR plan and applied to the IC_cyclo_ readings during the open-loop beam monitoring. The corrected MU readings from IC_cyclo_ will be recorded in the UHDR machine log files and extracted after delivery to correct for variabilities in the delivered MU.

### 2.2. mDs and fDs

**Figure 1.**
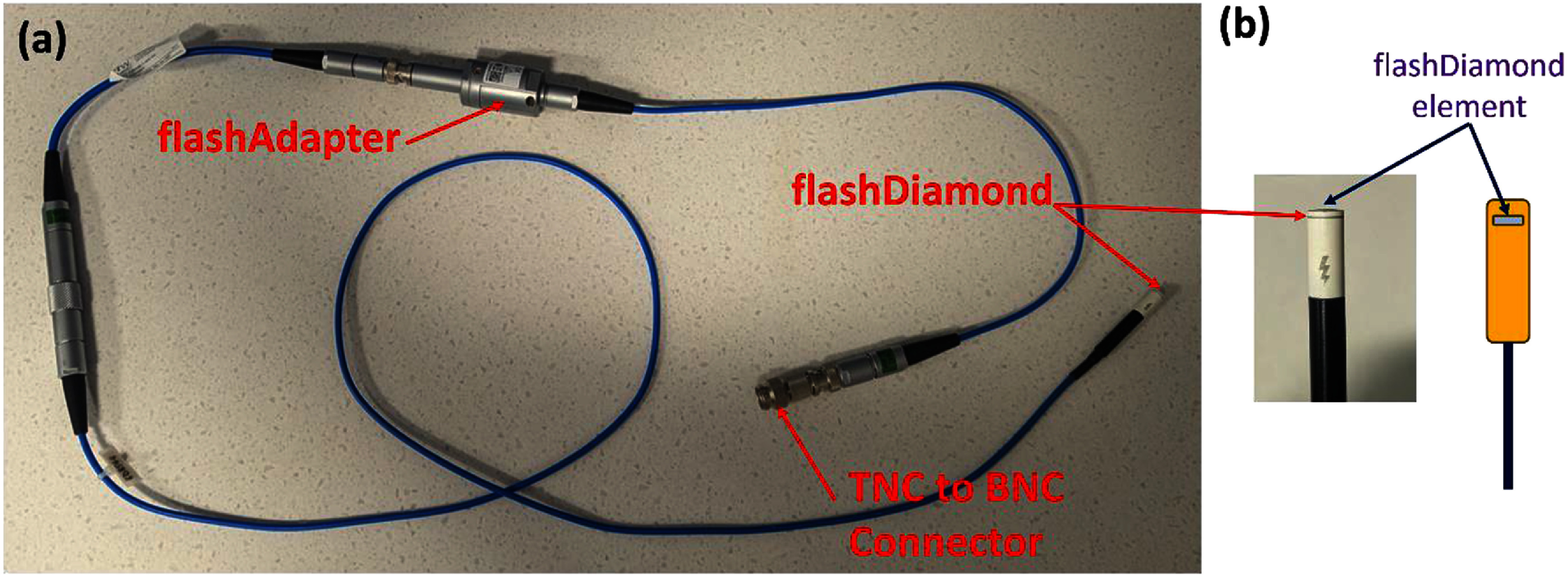
Photographs of the detector setup containing fD along with the associated flashAdapter. fD consists of an outer physical construction that closely resembles mD and its sensitive element consists of a small cylindrical disk that is embedded at its tip (figure (b)). fD is connected to the flashAdapter in series via a BNC connection and a further BNC-to-TNC adaptor was used to connect the detector to the associated electrometer. Please note that fD’s sensitive element is a cylinder of 0.06 cm radius and 0.0001 cm thickness and thus, have a nominal volume of 1.13 × 10^−6^ cm^3^.

Table 1 contains a summary of the various semiconductor detectors that were investigated within this study. Razor Nano was included to assess the small-field performance of the prototype fD detector relative to comparable ion chamber. mDs and Razor diodes were included to compare the relative accuracy of absolute proton UHDR dosimetry to all other commercially available semiconductor detectors. As seen, the sensitive volume of fD (as provided by the manufacturer) is significantly smaller than their mD counterparts to reduce the likelihood of charge saturation effects. fD (figure 1) also contains an associated flashAdapter that will be connected in series. The main purpose of the flashAdapter is to convert a sharp impulse signal from fD charge measurements into a square wave to reduce the likelihood of electrometer readout saturations without perturbing the magnitude of the total charge measured. This will be investigated by irradiating fDs with and without the flashAdapter under CONV and UHDR protons.

**Table 1.** Available technical specifications of the various ionization chambers and semiconductor detectors that were investigated within this study based on published literature and manufacturer’s provided specifications (Cavallone *et al* 2022). Please note that the electrode spacings for Razor Nano refer to the radius of the inner electrode. Razor Nano was included for small-field measurements.

Chamber model	Type	Diameter (cm)	Sensitive volume (cm^3^)	Electrode spacing (cm)	Operating voltage (V)
PPC05	Plane-parallel	0.99	0.046	0.06	±500
Razor Nano	Spherical	0.2	0.003	0.05	±400
mD	Disk	0.22	0.003 80	N/A	0
fD	Disk	0.12	1.13 × 10^−6^	N/A	0
Razor diode	Disk	0.06	6 × 10^−6^	N/A	0

### 2.3. Cross calibration protocol

**Figure 2.**
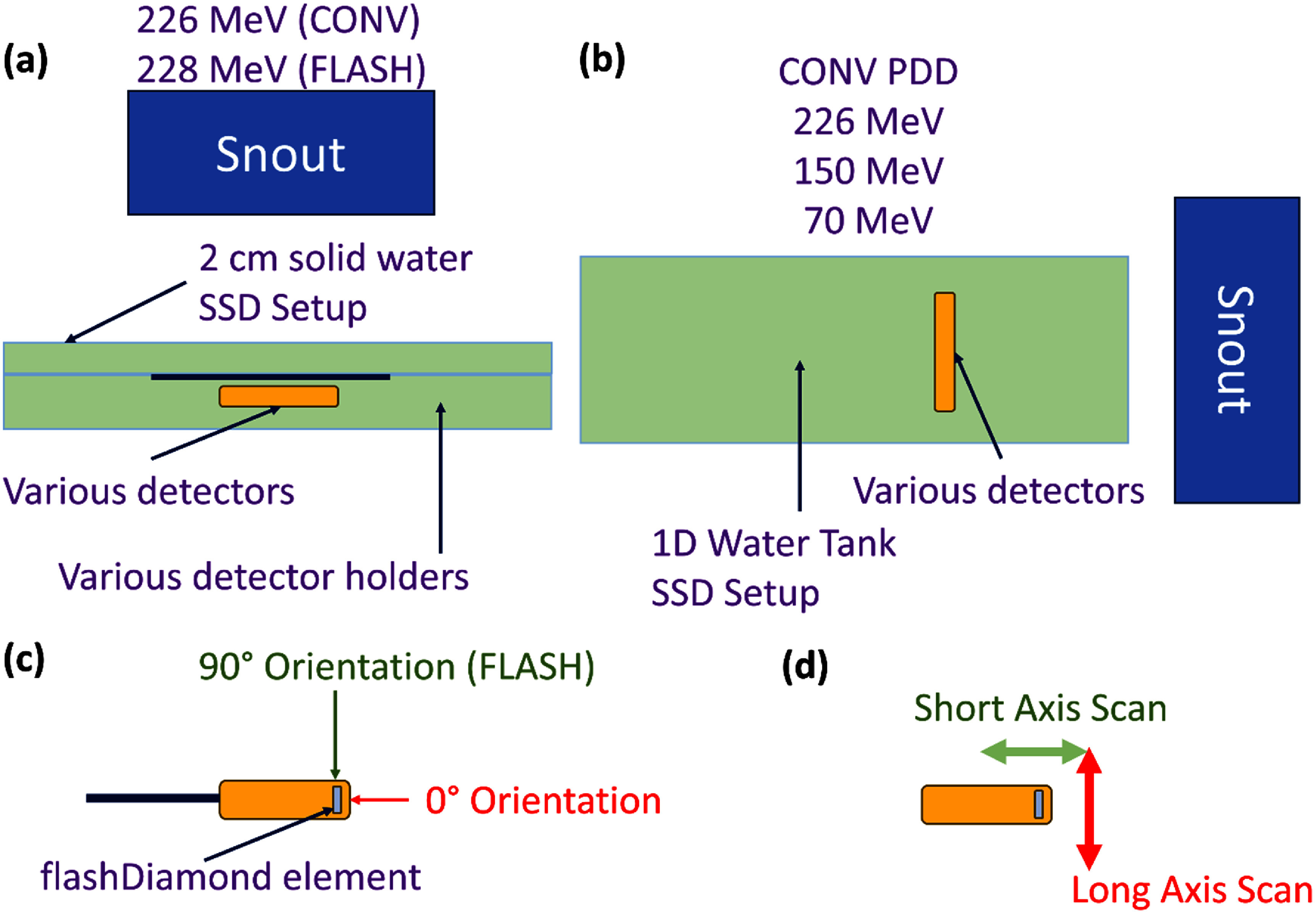
Illustrations of the various irradiation geometries that were used to characterize all detectors within UHDR and CONV proton beams. An (a): SSD setup at gantry 0° was generally used except for acquiring (b): PDDs. (c): fD and diode detectors were oriented in the 90° orientation for all UHDR and CONV proton irradiations with the exception of PDD measurements (0° orientation). (d): fD profile measurements were obtained over both crystal orientations.

As the beam optics of the proton synchrocyclotron has been tuned to deliver UHDR protons at a gantry of 0° for small animal irradiations, we cross-calibrated all of our detectors (table 1) within a similar setup (figure 2(a)) under conventional proton beams against a PPC05 PPCIC with an ADCL calibration coefficient of 59.23 cGy nC^−1^. The geometry that is depicted in figure 2(a) corresponds to an SSD setup within a solid water phantom (RW3 Slab Phantom, PTW dosimetry) of water equivalent thickness (WET) 1.033 at a solid water depth of 2.0 cm (2.066 cm WET). The PPC05 detector was housed within an RW3-PPC05-Adapterplate which consists of a cylindrical hollow cutout with no additional solid water buildup. The mD, fD and Razor diode detector were housed within a custom 3D printed resin holder (0.7 cm thickness, 1.09 WET) that will be housed within the RW3-PPC05-Adapterplate which will confer a total WET depth of 2.829 cm. The calibration proton beam consists of a 3 × 3 cm^2^ spot map consisting of 49 spots of 0.5 cm spacing delivered at a single 226 MeV energy to mimic the actual spot maps that would be delivered during UHDR proton irradiations. Correction factors were also applied to account for the additional 0.9% dose delivered to the semiconductor detectors due to their different WET as compared to the PPC05 detector during cross-calibrations. These correction factors were derived from treatment planning system (TPS) calculations (RayStation 2023B, RaySearch Laboratories, Stockholm, Sweden).

For all the semiconductor detectors, we derived cross-calibration factors using equation (1). The absolute proton doses to water were initially determined from PPC05 and thereafter corrected to account for the different WET thicknesses of the solid water irradiation geometries for the semiconductor detectors. Finally, the cross-calibration factor along with the beam quality factor was determined by dividing the WET-corrected absolute proton doses with the measured charges from the semiconductor detectors. Charge measurements were performed using an IBA DOSE 1 electrometer (Louvain-la-Neuve, Belgium) with an ADCL calibration coefficient of unity. Other than the PPC05 which was held at a bias voltage of +500 V, no bias voltages were applied to the semiconductor detectors. The beam quality factor ${k_{Q,{Q_0}}}$ that was used for PPC05 was 1.003. At CONV dose rates, the *P*_pol_ and *P*_ion_ of the PPC05 detector were found to be 1.0045 and 1.0026 respectively,

\begin{align*}{\left[ {{N_{D,w,{Q_0}}} \times {k_{Q,{Q_0}}}} \right]^{\prime}} = \frac{{{D_{w,Q}}}}{{\left[ {M_Q^{\prime}} \right]}} = \frac{{\left[ {{N_{D,w,{Q_0}}} \times {M_Q} \times {k_{Q,{Q_0}}}} \right]}}{{\left[ {M_Q^{\prime}} \right]}}\end{align*}

where ${\left[ {{N_{D,w,{Q_0}}} \times {k_{Q,{Q_0}}}} \right]^{\prime}}$ refers to the cross-calibration factor for the various detectors, ${D_{w,Q}}$ refers to the absorbed proton dose, $\left[ {M_Q^{\prime}} \right]$ refers to the corrected charge readings for the various detectors and $\left[ {{N_{D,w,{Q_0}}} \times {M_Q} \times {k_{Q,{Q_0}}}} \right]$ refers to the product of the PPC05’s ADCL calibration coefficient, corrected charge and beam quality factor respectively.

### 2.4. Characterization within conventional proton beams

Within the cross-calibration irradiation geometry (figure 2(a)) and at CONV proton dose rates, fD’s linearity and dose rate dependences were obtained. Dose linearity was established by delivering the CONV calibration proton spot maps (section 2.3) at varying CONV proton MUs per spots and measuring the charges collected from fD. Dose rate dependences at CONV dose rates were investigated by delivering the calibration proton field with an increasing amount of layer repainting while keeping the total MUs delivered constant. These measurements were performed at 3 different CONV proton energies (220 MeV, 150 MeV, 70 MeV) to establish any potential proton energy dependences.

In addition, within the irradiation geometry within figure 2(a), fD’s ability to measure profiles, i.e. lateral partial volume averaging artifacts, was investigated by shifting the robotic couch in millimeter increments and re-delivering the calibration CONV proton field. Within the solid water, fD would be irradiated in the 90° orientation (figure 2(c)) and profiles were obtained along both the long and short axes of fD’s active volume (figure 2(d)).

Potential LET dependences of fD were investigated using a different irradiation geometry (figure 2(b)) for the measurement of proton PDDs using a motorized 1D water tank (IBA Blue Phantom PT). A custom detector holder was 3D printed to secure fD to the motorized arm and it will be irradiated in the 0° orientation (figure 2(c)) using a 5 × 5 cm^2^ CONV proton spot map at 3 proton energies: 226 MeV, 150 MeV and 70 MeV. This process was repeated for mD and Razor Diode detectors to assess the differences in their LET responses.

### 2.5. Responses within UHDR proton beam

UHDR irradiations were performed using the irradiation geometry that is depicted in figure 2(a). The UHDR proton field was generated using maximum energy transmission proton beams which were made to scan a field similar to the previously used proton calibration field at CONV dose rates. The PPC05’s UHDR *P*_ion_ and *P*_pol_ were determined to be 1.0042 and 1.0097 respectively and were used to determine the corrected charge for the calculation of the absolute proton UHDR doses. Once this is done, the accuracy of fD relative to the PPC05’s response (ground truth) was assessed by repeating the measurements. Due to the differences in the detectors’ sensitivities, the FLASH MUs per spot were scaled up for fD irradiations to achieve similar SNRs between the detectors. We encountered slight fluctuations in the actual FLASH MUs delivered due to the open-loop delivery. To account for this, machine log files were obtained for each irradiation and were used to correct for the fluctuations of the MU delivered from their nominal values.

In addition, the pulsed nature of our UHDR proton beam was characterized by experimentally measuring the DPP of the pulsed beam. This was achieved by delivering a known number of proton pulses at UHDRs to a single spot on a radiochromic film (5 MU per pulse, 129.5 nA proton current at isocenter) at the highest dose rate. Absolute film dosimetry was then performed and the mean dose to a central 0.5 × 0.5 cm^2^ square region-of-interest (ROI) of the single spot UHDR irradiation was determined from an average of 3 film readings. The DPP was subsequently determined by dividing this mean central ROI dose with the number of pulses delivered.

### 2.6. Small field proton UHDR dosimetry

**Figure 3.**
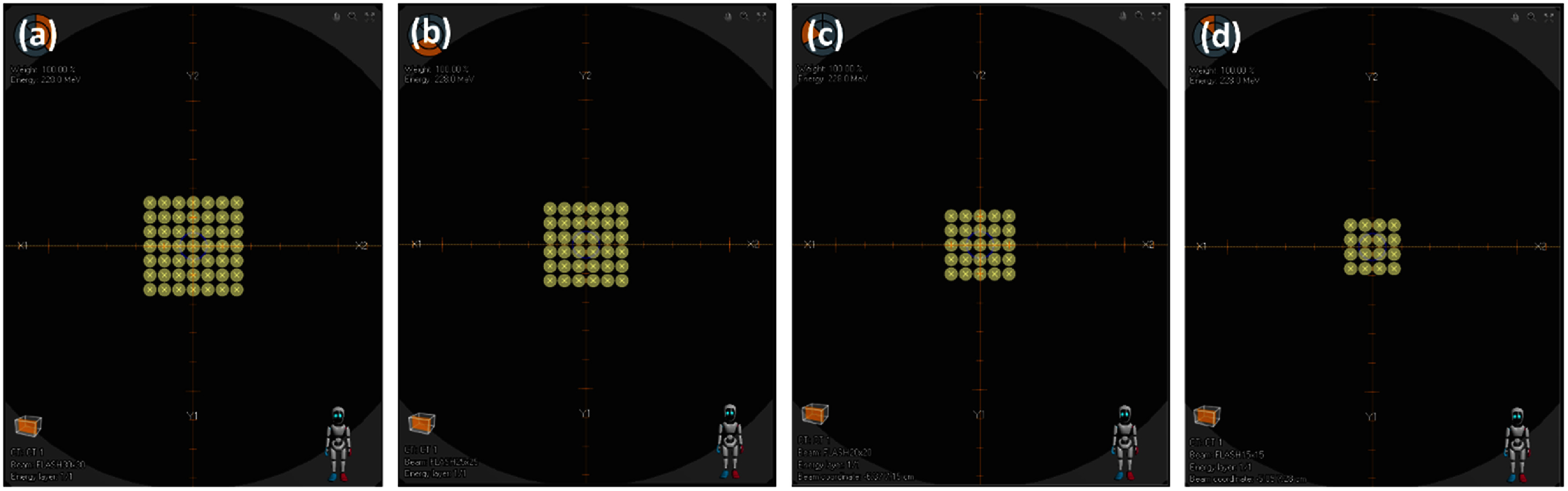
UHDR scanning proton spot maps for the (a): 3 × 3 cm^2^ (b): 2.5 × 2.5 cm^2^ (c): 2 × 2 cm^2^ and (d): 1.5 × 1.5 cm^2^ proton field sizes. Each spot map contains spots that are separated by 0.5 cm in the beam’s coordinates to result in (a): 7 × 7, (b): 6 × 6, (c): 5 × 5 and (d): 4 × 4 spot maps respectively. The spots were scanned in a raster-scanning pattern for the minimization of the delivery time. All spots had equivalent MUs or proton pulses. All UHDR absolute dose measurements were performed for the (a): 3 × 3 cm^2^ spot map with the (b)–(d): smaller field sizes used to assess the detectors’ (Razor Nano and fD) responses in small proton fields.

Finally, we assessed the ability of fD to perform accurate proton small field dosimetry under UHDR conditions (figure 3). UHDR fields ranging from 3 × 3 cm^2^ to 1.5 × 1.5 cm^2^ were delivered by decreasing the spot maps from 7 × 7 to 4 × 4. This process was repeated for mD. As the PPC05 was clearly too large for a 1.5 × 1.5 cm^2^ measurement, we used a Razor Nano ion chamber for comparisons with ion chamber detectors. These small field measurements were supplemented with radiochromic film dosimetry (Gafchromic EBT-XD, Ashland) and RayStation TPS calculations.

## 3. Results

### 3.1. Faraday cup measurements

The repeatability of the UHDR proton irradiations was assessed by delivering a single spot (28 pulses, 5 MU per pulse, 140 total MU) to the Faraday cup at isocenter and these measurements were repeated five times and presented in table 2. There can be a relative difference between the requested and delivered FLASH MU by up to 1.3%. After correcting for these uncertainties, there is a 0.80% standard deviation for repeated UHDR deliveries as compared to 0.120% standard deviation at CONV dose rates. After correcting for the intrinsic charge collection efficiency of the Faraday cup and given the pulse repetition rate of 1000 Hz, the average nozzle current at the isocenter for the current UHDR configuration was calculated to be 129.5 nA with the instantaneous current calculated to be approximately 12.95 *μ*A from the approximate pulse width of 10 *μ*s at extraction.

**Table 2.** Faraday cup measurements of a single UHDR proton spot consisting of 28 pulses, 5 MU per pulse and 140 total nominal MU.

Runs	MU delivered	Output	Raw charge (nC)	Normalized charge (nC)
1	140.881	1.006	3.508	3.486
2	141.185	1.008	3.439	3.410
3	140.808	1.006	3.482	3.462
4	141.787	1.013	3.507	3.463
5	141.167	1.008	3.488	3.459

Absolute radiochromic film dosimetry of the single spot UHDR delivery was performed for 28, 40 and 56 pulses at the highest dose rate for a total of 140, 200 and 280 FLASH MUs. Experimental doses were determined to be 898, 1305 and 1850 cGy respectively which corresponded to an experimentally-determined DPP value of 32.6 ± 0.5 cGy per pulse.

### 3.2. Detector cross calibration factors

The results of the various cross-calibration coefficients are presented in table 3. Despite the lowered sensitivity of fD, it was found to have a percentage standard deviation of 0.418% which is lower than the repeatability of the UHDR deliveries. Error analyses were performed using the TRS-398 published uncertainty budget for PPICs. However, we used an updated beam quality uncertainty factor of 1.2% from recent publications (Palmans *et al* 2022). There is an intrinsic ±1.67% uncertainty in the determination of the absorbed proton dose during cross-calibration as well as the intrinsic fluctuation of the PPC05’s response over 10 repeated readings.

**Table 3.** Results of the cross-calibration factors derived across the various detectors investigated within this study. The factors within solid water have been corrected to account for the differences in proton doses delivered within this geometry due to differences in the WET depth within the solid water. The percentage standard deviations were obtained from a set of 10 repeated readings. PPC05 had an ADCL calibration coefficient of 59.23 cGy nC^−1^. According to the manufacturer, the nominal response of fD is (0.0025 $ \pm $ 0.1) nC cGy^−1^ in a ^60^Co beam.

Detector	Cross-calibration factor (cGy nC^−1^)	Standard deviation (%)	Total uncertainty (%)
mD	95.35	0.162	1.678
fD	613.29	0.418	1.722
Razor diode	28.62	0.326	1.702

fD had a relatively higher percentage standard deviation relative to mDs and the Razor Diodes due to its lowered sensitivity. Its cross-calibration factor of 613.29 cGy nC^−1^ is within the same order of its nominal response under a ^60^Co beam as reported by the manufacturer. While the cross-calibration factors at CONV dose rates were obtained with the flashAdapter to mimic the actual UHDR measurement conditions, we verified that the total charges collected were unaffected (within ±0.5%) by the presence of the flashAdapter.

### 3.3. Response of fDs under conventional protons

**Figure 4.**
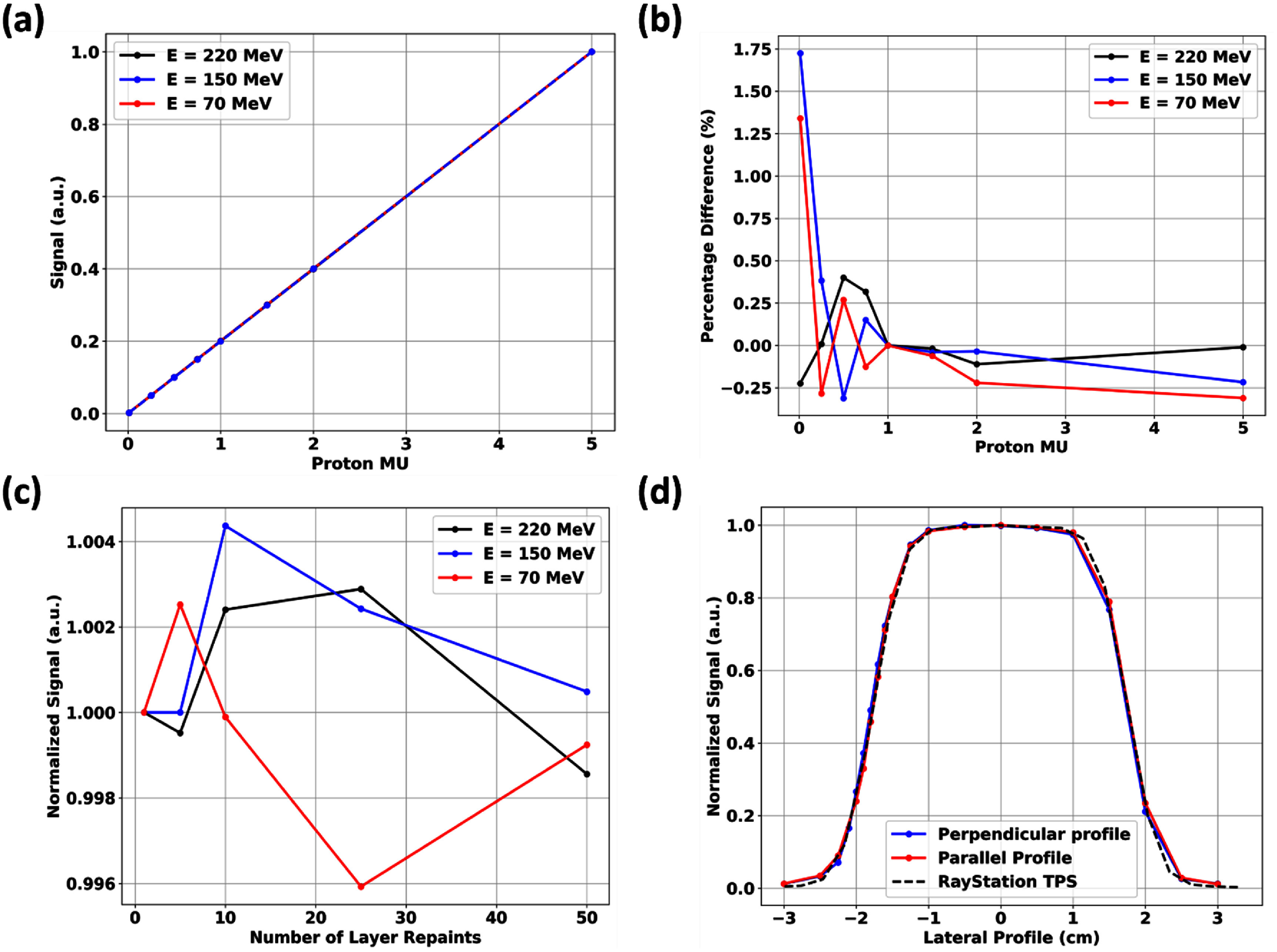
Various responses of fD with the attached flashAdapter under conventional proton irradiations utilizing a 3 × 3 cm^2^ conventional proton beam. (a): Plot of the linearity of fD responses under differing proton MUs at 220 MeV, 150 MeV and 70 MeV proton irradiations. (b): Plot of the percentage deviations from the expected linear response. (c): Plot of the relative responses of fD under conventional proton beams with layer repainting (preliminary test of the dose rate dependences of fD) normalized to no repainting delivery. (d): Profile scans of a 3 × 3 cm^2^ conventional proton beam along the long and the short fD axes (figure 2(d)).

Figures 4(a) and (b) show the response linearity of fD across a wide proton dose latitude. fD is generally linear within ±0.5% of the expected values with relatively larger deviations at the minimum MU settings of the proton machine of 0.0012 MU. Figure 4(c) shows the dose-rate independence (±0.5%) of fD under multiple layer repainting at conventional proton dose rates. In addition, there is very little evidence of proton energy dependences of fD.

**Figure 5.**
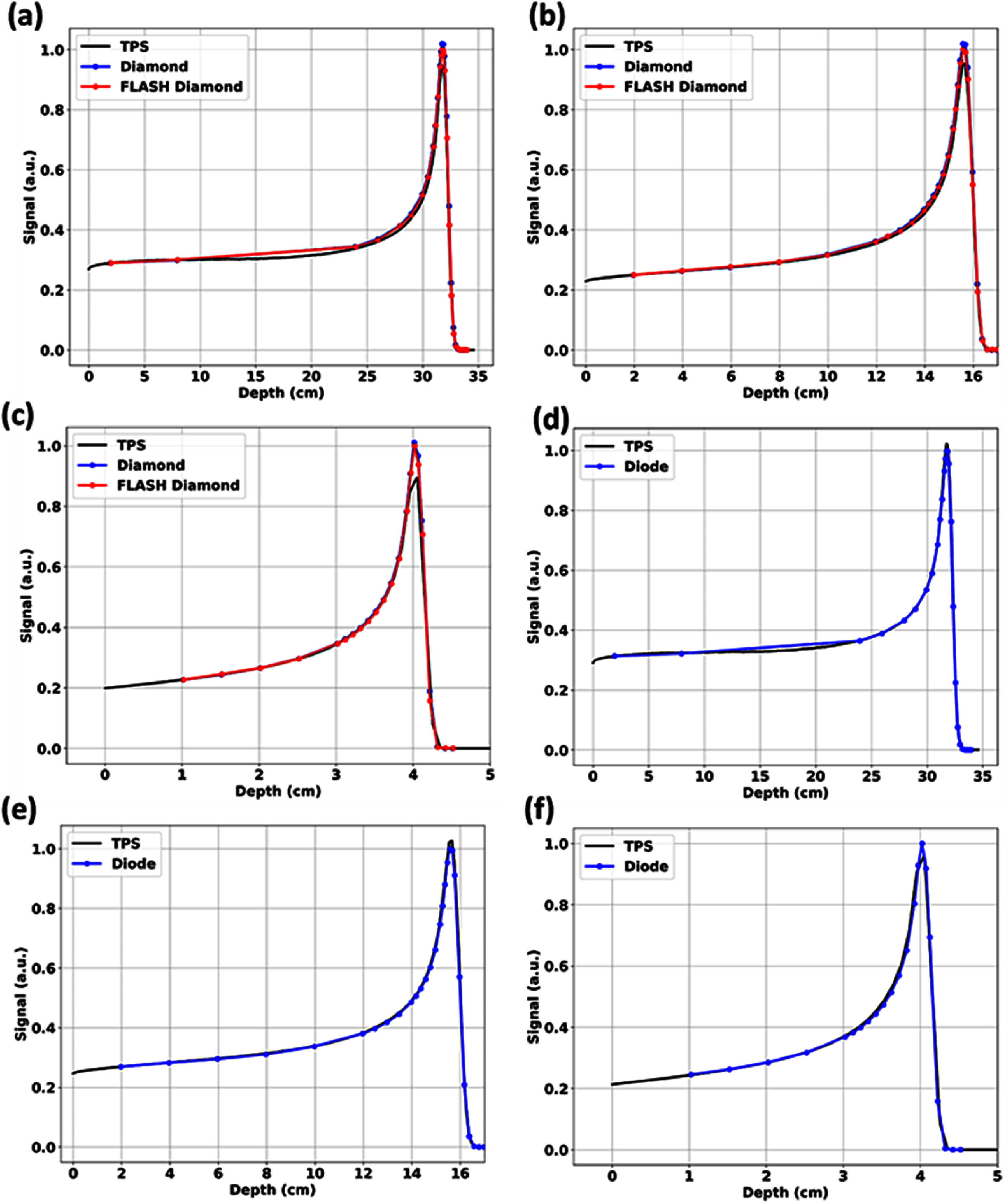
Proton PDDs obtained (figure 2(b)) for (a)–(c): both diamond detectors and (d)–(f): diode detector for 226 MeV, 150 MeV and 70 MeV proton beams where all detectors were oriented in the 0° parallel detector orientation (figure 2(c)) relative to the 5 × 5 cm^2^ conventional proton beam. Diamond detectors exhibited similar detector responses while the diode detector exhibited water-equivalent detector responses.

The lateral profiles measured by fD did not exhibit any partial volume artifacts even along its long-axis and it closely matched TPS-calculated profiles of the calibration 3 × 3 cm^2^ field as seen in figure 4(d). fD did not exhibit any significant LET effects as seen in PDD measurements in figure 5. It was found to match the response of mD. However, for very sharp Bragg peaks or small distal penumbras such as for lower proton energies with minimal range straggling, there is a slight over-response artifact for both diamond detectors. This is less apparent for the Razor Diode detectors, which also did not exhibit any LET effects under proton irradiations.

**Figure 6.**
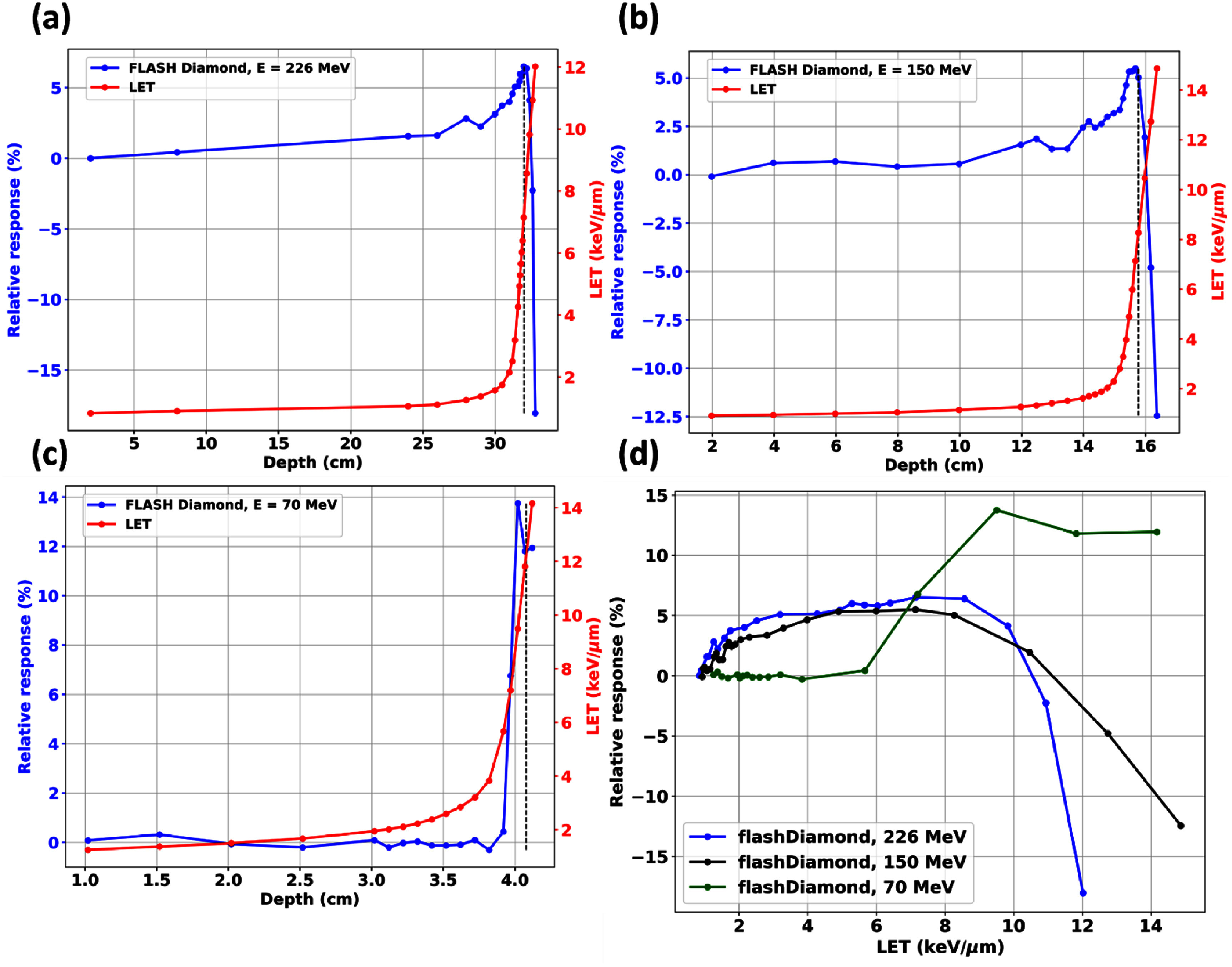
Plots of the percentage deviations of fD’s responses relative to proton PDDs of conventional proton beams (226 MeV, 150 MeV and 70 MeV). The dose-averaged LET was superimposed demonstrating minimal LET dependence. There are similar mD and fD over-responses at the proton Bragg peak. The vertical black dashed line corresponded to the locations of the distal 90% (R90) of the Bragg peak. (d): Plot of relative response vs LET demonstrating their non-correlation.

Figure 6 shows plots of the relative responses of fD with depth and the TPS-calculated dose-averaged LET (LET_d_) values were overlaid for reference. As seen, there is generally a maximum over-response of approximately 5.49%–6.51% at the Bragg peak at high to intermediate proton energies. For the sharper Bragg peaks that were encountered at low energies, this over-response was found to be as high as 13.7%. The behaviors of the relative responses were similar between the diamond detectors (figure 5) while the diode detectors more closely matched the actual TPS calculated proton PDDs (figure 5). The relative responses of these semiconductor detectors were found to be LET independent; for example, the relative responses of fDs at similar LET_d_ values of 2 keV *μ*m^−1^ are clearly different for different energies.

### 3.4. Absolute proton UHDR dosimetry

The results of the absolute proton dose calculations for all semiconductor detectors are shown in table 4. Except for the Razor Diode, all chambers were within ±1% of the PPC05 readings. Despite the pulsed nature of the proton UHDR beam and its high instantaneous current, current mDs did not exhibit any charge saturation effects at the current proton UHDR configuration. Further investigations showed that fD responded without charge saturation even when the flashAdapter was not used.

**Table 4.** Results of the absolute UHDR proton dose calculations from the previously derived cross-calibration correction factors and UHDR *P*_pol_ and *P*_ion_ correction factors for PPC05. The total MUs were scaled to account for the lowered sensitivities of fD and achieve a comparable signal-to-noise-ratio (SNR) for all UHDR irradiations. The experimental average dose/MU per spot was calculated from all detectors to be 47.8 ± 0.9 cGy. Razor diode experimental measurements were excluded from the average as it was clearly an outlier.

Detector	Total MU	Dose (cGy)	Dose/MU per spot (cGy)	Percentage difference (%)	
				PPC05	Group
PPC05	980	957.79	47.89	N/A	0.14
mD	980	950.39	47.52	−0.77	−0.64
fD	2940	2870.05	47.83	0.78	0.01
fD without flashAdapter		2883.34	48.06	0.35	0.49
Razor diode	980	994.06	49.70	3.79	3.92

The average dose per MU per spot was determined to be 47.8 ± 0.9 cGy from all detectors except the Razor diode detector which was clearly an outlier. The uncertainty budget was determined to be 1.9% which accounts for the ±1.7% systematic uncertainty from the CONV absolute proton dose measurements for cross-calibrations, ±0.8% random uncertainty from the UHDR delivery repeatability and ±0.3% uncertainty from the detector response repeatability.

### 3.5. Small field pencil-beam-scanning (PBS) proton UHDR measurements

**Figure 7.**
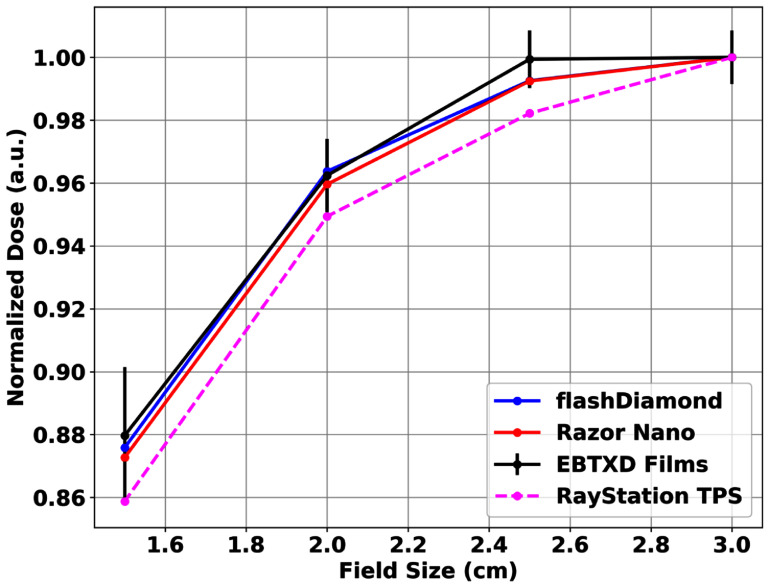
Relative responses of fDs at small proton UHDR field sizes (figure 3) along with Razor Nano ion chambers and EBT-XD radiochromic film measurements. Expected values from RayStation TPS calculations were superimposed for reference. Error bars present represent the variance of repeated film measurements which accounted for the higher experimental variability of film absolute dosimetry. Normalization was performed at 3 × 3 cm^2^.

Figure 7 shows a plot of the relative responses of fD under UHDR beams of different fields (figure 3). Overlaid are similar plots of other comparable detectors, film measurements and RayStation TPS dose calculations. All detectors investigated closely responded within ±1% of one another and agreed with TPS calculations within ±2%. Error bars represented the percentage standard deviation of repeated film readings. Experimental uncertainties were dominated by the repeatability of the UHDR beam of approximately ±0.8% followed by the repeatability of the detectors’ response (table 3) and can be estimated to be ±1.0%.

### 3.6. UHDR dose rate calculations

For all UHDR spot maps delivered (figure 3), we verified that there were no slewing dead times between pulses. From the machine delivery logs, we calculated the average dose rates to be 49.0, 66.2, 92.6 and 131.5 Gy s^−1^ for 3.0 × 3.0, 2.5 × 2.5, 2.0 × 2.0 and 1.5 × 1.5 cm^2^ field sizes respectively. RayStation TPS calculations showed a voxelwise maximum dose rate of 330 Gy s^−1^ at the detector. We delivered all UHDR irradiations at the maximum nozzle current of 129.5 nA or 5 MU per pulse.

## 4. Discussion

In previous works with pulsed electron UHDR deliveries, it was shown that mD and diode detectors exhibit a nonreversible charge saturation phenomenon beyond a DPP value of 15.0 cGy per pulse (Di Martino *et al* 2020, Kranzer *et al* 2022). However, despite our UHDR proton beams having a DPP value that is double of threshold values in literature, there were no charge saturations within commercially available mDs. These differences are likely due to the different magnitudes of the instantaneous dose rates which are dependent on the intra-pulse temporal structures; the pulse extraction window of the S2C2 synchrocyclotron is approximately 10 *μ*s as compared to the pulse widths of electron UHDR machines which are typically below 4 *μ*s (Ashraf *et al* 2024, Tessonnier *et al* 2024). To our knowledge, this is the first investigation of fDs within a pulsed proton UHDR beam and while it would be useful and interesting to determine the DPP threshold to better understand the suitability of mD for UHDR dosimetry, the S2C2 synchrocyclotron’s DPP values could not be currently substantially increased beyond its current value. The current UHDR configuration of the S2C2 synchrocyclotron of 129.5 nA proton current at isocenter (approximately 12.95 *μ*A instantaneous proton current) is generated at a cyclotron Dee voltage that is at 98.5% of its present engineering limit. However, the present S2C2’s UHDR configuration has not been finalized and there are ongoing developmental works at IBA on further increasing the beam current and DPP values. It is highly likely that fDs will be more ideal than mDs for future UHDR configurations with DPPs higher than the current value.

The magnitudes of the collected charges from fD irradiations were not perturbed by the flashAdapter as assessed within CONV protons. However, as the DPP values of our proton beams did not result in any noticeable electrometer saturations, the efficacy of the flashAdapter could not be assessed within this study. Future works will involve the investigation of its efficacy in mitigating electrometer-related saturations in higher proton DPP environments.

Potential uncertainties from the open-loop delivery were accounted for by correcting deviations between the nominal and delivered MUs using log files. Systematic deviations greater than 2% manifested for nozzle currents below 12.5 nA or MU per pulse values below 0.5 MU. Larger systematic deviations at lower nozzle currents manifest from rounding errors in the number of delivered MUs per pulse over a greater number of pulse and the increased relative error in these rounding errors to nominal MU per pulse values. Prior to this study, we assessed the accuracy of these log files by delivering a single 200 MU spot to a Faraday cup at isocenter at various nozzle currents (12.5–129.5 nA). The resultant corrected experimental Faraday cup readings were within ±0.5% of the group’s mean demonstrating the correlation between the delivered MUs, log file records and experimental Faraday cup readings. The error estimate of the UHDR delivery repeatability of ±0.80% was obtained after performing log-file correction and thus necessarily includes any uncertainties from the log file records.

Compared with mD, fD is relatively insensitive. Its nominal response according to the manufacturer is approximately 0.0025 nC cGy^−1^ as compared to 0.01 nC cGy^−1^ for mDs.^63^ When calibrated with protons, our mD showed a sensitivity response of 95.35 cGy nC^−1^ (0.01049 nC cGy^−1^) that is close to its nominal response while fD’s sensitivity of 613.29 cGy nC^−1^ (0.00163 nC cGy^−1^) is much less insensitive relative to its nominal response. We note that the nominal volume of the fD’s sensitive element is 29.7% of that of the corresponding nominal volume of mD and this value is closer to the expected differences in their sensitivities. As such, these discrepancies are likely attributed to the variabilities in their inner constructions of their sensitive elements. Our fD has a sensitivity that is 15.5% of mD’s sensitivity and 9.65% of the PPC05’s sensitivity. As such, they are ideal for high dose measurements in UHDR irradiations. When cross-calibrated in CONV proton beams, a proton dose of 890 cGy was delivered repeatedly to fD which resulted in an average measured charge of 1.455 ± 0.006 nC (0.4% percentage standard deviation). Compared to fD, equivalent percentage standard deviation values were achieved for the rest of the detectors (PPC05, mD, Razor diode) at 20% of the CONV proton doses that were delivered to fD. This was the reason why the doses delivered to fD were scaled up (table 4) to achieve comparable SNRs with the rest of the detectors investigated.

The fD’s small sensitive element makes it ideal for small field dosimetry which is relevant for preclinical and radiobiological UHDR irradiations. fD responded within ±1% as EBT-XD absolute film dosimetry measurements and Razor Nano detectors down to field sizes of 1.5 × 1.5 cm^2^. In addition, it does not exhibit substantial lateral penumbral broadening when scanned along both its long- and short-axes which makes it ideal for small-field profile measurements. Within our institution, we envision the routine use of fDs during UHDR irradiations as a consistency check of actual UHDR doses delivered to small animals like C57BL/6 mice. This is meant to complement post-irradiation log file analyses to correct for potential uncertainties from open-loop beam deliveries. Specifically, we envision their use as exit dosimeters for transmission UHDR irradiations with an initially acquired relative baseline prior to actual irradiations. Such UHDR irradiations often involve the delivery of doses above 1000 cGy using field sizes that are as small as 1.1 cm (Levy *et al* 2020, Kim *et al* 2021, 2024, Evans *et al* 2022, Zhang *et al* 2023, Cao *et al* 2024, Bell *et al* 2025) for which fD is well-suited for. fDs were LET and energy dependent; their responses were generally water-equivalent under most of the proton PDD despite the increasing LET_d_ values with depth. This agrees with previous studies that were performed in literature which showed similar LET independence within proton beams (Rossomme *et al* 2016). Rather, the locations of these over-response seem to be correlated to areas of steep spatial dose gradients. We note that the proton PDDs were obtained along the short axis of both diamond detectors’ sensitive elements which have a thickness of approximately 1 *μ*m and hence, of a much finer spatial resolution than the 0.1 cm dose calculation resolution of clinical RayStation TPS calculations. As such, TPS calculations are likely to be coarse-grained to 0.1 cm along the beam’s direction which will lead to an under-scoring of the proton doses under the pristine Bragg peak. We also note that this over-response phenomenon was also recently reported during the profile measurements of proton minibeams during which over-responses that were greater than 10% were reported (Sotiropoulos and Prezado 2022). Nevertheless, we do not envision these spatial effects to affect the accuracy of UHDR dosimetry measurements nor the radiobiological conclusions or outcomes of UHDR irradiations. Instead, their water-equivalence and LET-independence under proton irradiations make these detectors ideal for routine uses.

## 5. Conclusion

Novel fDs can be used for pulsed UHDR PBS proton dosimetry with DPP values of 32.6 cGy. Their small sensitive elements make them ideal for small-field dosimetry and/or UHDR profile measurements at the cost of reduced dose sensitivities which are offset by the relatively higher doses typically delivered for UHDR irradiations. However, current mDs do not saturate within the pulsed UHDR beam of the current S2C2 synchrocyclotron UHDR configuration and therefore can still be used for routine UHDR dosimetry. fDs may be better indicated for future UHDR configurations utilizing higher proton currents and DPP values. Future works will involve a more comprehensive study of the DPP linearity effects and the threshold beyond which the use of fDs are necessary.

## Data Availability

All data that support the findings of this study are included within the article (and any supplementary information files).

## References

[pmbae023bbib1] Ashraf M R (2024). Multi-institutional audit of FLASH and conventional dosimetry with a 3D printed anatomically realistic mouse phantom. Int. J. Radiat. Oncol. Biol. Phys..

[pmbae023bbib2] Atkinson J, Bezak E, Le H, Kempson I (2023). The current status of FLASH particle therapy: a systematic review. Phys. Eng. Sci. Med..

[pmbae023bbib3] Bell B I (2025). Whole abdominal pencil beam scanned proton FLASH increases acute lethality. Int. J. Radiat. Oncol. Biol. Phys..

[pmbae023bbib4] Cao N (2024). Preclinical ultra-high dose rate (FLASH) proton radiation therapy system for small animal studies. Adv. Radiat. Oncol..

[pmbae023bbib5] Cavallone M, Goncalves Jorge P, Moeckli R, Bailat C, Flacco A, Prezado Y, Delorme R (2022). Determination of the ion collection efficiency of the Razor Nano Chamber for ultra-high dose-rate electron beams. Med. Phys..

[pmbae023bbib6] Chow J C L, Ruda H E (2024). Mechanisms of action in FLASH radiotherapy: a comprehensive review of physicochemical and biological processes on cancerous and normal cells. Cells.

[pmbae023bbib7] Darafsheh A, Hao Y, Zhao X, Zwart T, Wagner M, Evans T, Reynoso F, Zhao T (2021). Spread-out Bragg peak proton FLASH irradiation using a clinical synchrocyclotron: proof of concept and ion chamber characterization. Med. Phys..

[pmbae023bbib8] Darafsheh A, Hao Y, Zwart T, Wagner M, Catanzano D, Williamson J F, Knutson N, Sun B, Mutic S, Zhao T (2020). Feasibility of proton FLASH irradiation using a synchrocyclotron for preclinical studies. Med. Phys..

[pmbae023bbib9] Daugherty E C (2024). FLASH radiotherapy for the treatment of symptomatic bone metastases in the thorax (FAST-02): protocol for a prospective study of a novel radiotherapy approach. Radiat. Oncol..

[pmbae023bbib10] Deffet S, Hamaide V, Sterpin E (2023). Definition of dose rate for FLASH pencil-beam scanning proton therapy: a comparative study. Med. Phys..

[pmbae023bbib11] Di Martino F (2020). FLASH radiotherapy with electrons: issues related to the production, monitoring, and dosimetric characterization of the beam. Front. Phys..

[pmbae023bbib12] Diffenderfer E S (2020). Design, implementation, and *in vivo* validation of a novel proton FLASH radiation therapy system. Int. J. Radiat. Oncol. Biol. Phys..

[pmbae023bbib13] Diffenderfer E S, Sorensen B S, Mazal A, Carlson D J (2022). The current status of preclinical proton FLASH radiation and future directions. Med. Phys..

[pmbae023bbib14] Esplen N, Mendonca M S, Bazalova-Carter M (2020). Physics and biology of ultrahigh dose-rate (FLASH) radiotherapy: a topical review. Phys. Med. Biol..

[pmbae023bbib15] Evans T, Cooley J, Wagner M, Yu T, Zwart T (2022). Demonstration of the FLASH effect within the spread-out Bragg peak after abdominal irradiation of mice. Int. J. Part. Ther..

[pmbae023bbib16] Fenwick J D, Mayhew C, Jolly S, Amos R A, Hawkins M A (2024). Navigating the straits: realizing the potential of proton FLASH through physics advances and further pre-clinical characterization. Front. Oncol..

[pmbae023bbib17] Gao H (2022a). Simultaneous dose and dose rate optimization (SDDRO) of the FLASH effect for pencil-beam-scanning proton therapy. Med. Phys..

[pmbae023bbib18] Gao H, Lin B, Lin Y, Fu S, Langen K, Liu T, Bradley J (2020). Simultaneous dose and dose rate optimization (SDDRO) for FLASH proton therapy. Med. Phys..

[pmbae023bbib19] Gao Y, Liu R, Chang C W, Charyyev S, Zhou J, Bradley J D, Liu T, Yang X (2022b). A potential revolution in cancer treatment: a topical review of FLASH radiotherapy. J. Appl. Clin. Med. Phys..

[pmbae023bbib20] Giannini N, Gadducci G, Fuentes T, Gonnelli A, Di Martino F, Puccini P, Naso M, Pasqualetti F, Capaccioli S, Paiar F (2024). Electron FLASH radiotherapy *in vivo* studies. A systematic review. Front. Oncol..

[pmbae023bbib21] Hageman E, Che P-P, Dahele M, Slotman B J, Sminia P (2022). Radiobiological aspects of FLASH radiotherapy. Biomolecules.

[pmbae023bbib22] Hughes J R, Parsons J L (2020). FLASH radiotherapy: current knowledge and future insights using proton-beam therapy. Int. J. Mol. Sci..

[pmbae023bbib23] Jolly S, Owen H, Schippers M, Welsch C (2020). Technical challenges for FLASH proton therapy. Phys. Med..

[pmbae023bbib24] Kacem H, Almeida A, Cherbuin N, Vozenin M-C (2022). Understanding the FLASH effect to unravel the potential of ultra-high dose rate irradiation. Int. J. Radiat. Biol..

[pmbae023bbib25] Kang M, Wei S, Choi J I, Lin H, Simone C B (2022). A universal range shifter and range compensator can enable proton pencil beam scanning single-energy Bragg peak FLASH-RT treatment using current commercially available proton systems. Int. J. Radiat. Oncol. Biol. Phys..

[pmbae023bbib26] Karsch L (2022). Beam pulse structure and dose rate as determinants for the flash effect observed in zebrafish embryo. Radiother. Oncol..

[pmbae023bbib27] Kim K (2024). FLASH proton radiation therapy mitigates inflammatory and fibrotic pathways and preserves cardiac function in a preclinical mouse model of radiation-induced heart disease. Int. J. Radiat. Oncol. Biol. Phys..

[pmbae023bbib28] Kim M M (2021). Comparison of FLASH proton entrance and the spread-out Bragg peak dose regions in the sparing of mouse intestinal crypts and in a pancreatic tumor model. Cancers.

[pmbae023bbib29] Kranzer R, Schuller A, Bourgouin A, Hackel T, Poppinga D, Lapp M, Looe H K, Poppe B (2022). Response of diamond detectors in ultra-high dose-per-pulse electron beams for dosimetry at FLASH radiotherapy. Phys. Med. Biol..

[pmbae023bbib30] Kroll F (2022). Tumour irradiation in mice with a laser-accelerated proton beam. Nat. Phys..

[pmbae023bbib31] Levy K (2020). Abdominal FLASH irradiation reduces radiation-induced gastrointestinal toxicity for the treatment of ovarian cancer in mice. Sci. Rep..

[pmbae023bbib32] Limoli C L, Vozenin M-C (2023). Reinventing radiobiology in the light of FLASH radiotherapy. Annu. Rev. Cancer Biol..

[pmbae023bbib33] Lin B, Gao F, Yang Y, Wu D, Zhang Y, Feng G, Dai T, Du X (2021a). FLASH radiotherapy: history and future. Front. Oncol..

[pmbae023bbib34] Lin B, Huang D, Gao F, Yang Y, Wu D, Zhang Y, Feng G, Dai T, Du X (2022). Mechanisms of FLASH effect. Front. Oncol..

[pmbae023bbib35] Lin Y, Li W, Johnson D, Prezado Y, Gan G N, Gao H (2024a). Development and characterization of the first proton minibeam system for single-gantry proton facility. Med. Phys..

[pmbae023bbib36] Lin Y, Li W, Wang A, Johnson D, Gan G N, Gao H (2024b). Comprehensive dosimetric commissioning of proton minibeam radiotherapy on a single gantry proton system. Front. Oncol..

[pmbae023bbib37] Lin Y, Lin B, Fu S, Folkerts M M, Abel E, Bradley J, Gao H (2021b). SDDRO-joint: simultaneous dose and dose rate optimization with the joint use of transmission beams and Bragg peaks for FLASH proton therapy. Phys. Med. Biol..

[pmbae023bbib38] Liu R (2023). An integrated physical optimization framework for proton stereotactic body radiation therapy FLASH treatment planning allows dose, dose rate, and linear energy transfer optimization using patient-specific ridge filters. Int. J. Radiat. Oncol. Biol. Phys..

[pmbae023bbib39] Lourenco A (2023). Absolute dosimetry for FLASH proton pencil beam scanning radiotherapy. Sci. Rep..

[pmbae023bbib40] Lyu Q, Neph R, O’Connor D, Ruan D, Boucher S, Sheng K (2021). ROAD: ROtational direct Aperture optimization with a Decoupled ring-collimator for FLASH radiotherapy. Phys. Med. Biol..

[pmbae023bbib41] Ma J, Lin Y, Tang M, Zhu Y-N, Gan G N, Rotondo R L, Chen R C, Gao H (2024a). Simultaneous dose and dose rate optimization via dose modifying factor modeling for FLASH effective dose. Med. Phys..

[pmbae023bbib42] Ma Y (2024b). Current views on mechanisms of the FLASH effect in cancer radiotherapy. Natl Sci. Rev..

[pmbae023bbib43] Mascia A E (2023). Proton FLASH radiotherapy for the treatment of symptomatic bone metastases: the FAST-01 nonrandomized trial. JAMA Oncol..

[pmbae023bbib44] Matuszak N, Suchorska W M, Milecki P, Kruszyna-Mochalska M, Misiarz A, Pracz J, Malicki J (2022). FLASH radiotherapy: an emerging approach in radiation therapy. Rep. Pract. Oncol. Radiother..

[pmbae023bbib45] McGarrigle J M, Long K R, Prezado Y (2024). The FLASH effect-an evaluation of preclinical studies of ultra-high dose rate radiotherapy. Front. Oncol..

[pmbae023bbib46] McManus M, Romano F, Lee N D, Farabolini W, Gilardi A, Royle G, Palmans H, Subiel A (2020). The challenge of ionisation chamber dosimetry in ultra-short pulsed high dose-rate very high energy electron beams. Sci. Rep..

[pmbae023bbib47] Montay‐Gruel P, Corde S, Laissue J A, Bazalova‐Carter M (2022). FLASH radiotherapy with photon beams. Med. Phys..

[pmbae023bbib48] Palmans H, Lourenco A, Medin J, Vatnitsky S, Andreo P (2022). Current best estimates of beam quality correction factors for reference dosimetry of clinical proton beams. Phys. Med. Biol..

[pmbae023bbib49] Patriarca A (2018). Experimental set-up for FLASH proton irradiation of small animals using a clinical system. Int. J. Radiat. Oncol. Biol. Phys..

[pmbae023bbib50] Rahman M (2021). Electron FLASH delivery at treatment room isocenter for efficient reversible conversion of a clinical LINAC. Int. J. Radiat. Oncol. Biol. Phys..

[pmbae023bbib51] Rossomme S, Denis J M, Souris K, Delor A, Bartier F, Dumont D, Vynckier S, Palmans H (2016). LET dependence of the response of a PTW-60019 microDiamond detector in a 62 MeV proton beam. Phys. Med..

[pmbae023bbib52] Schuler E, Acharya M, Montay‐Gruel P, Loo B W, Vozenin M-C, Maxim P G (2022). Ultra-high dose rate electron beams and the FLASH effect: from preclinical evidence to a new radiotherapy paradigm. Med. Phys..

[pmbae023bbib53] Sorensen B S, Kanouta E, Ankjaergaard C, Kristensen L, Johansen J G, Sitarz M K, Andersen C E, Grau C, Poulsen P (2024). Proton FLASH: impact of dose rate and split dose on acute skin toxicity in a murine model. Int. J. Radiat. Oncol. Biol. Phys..

[pmbae023bbib54] Sotiropoulos M, Prezado Y (2022). Radiation quality correction factors for improved dosimetry in preclinical minibeam radiotherapy. Med. Phys..

[pmbae023bbib55] Spruijt K (2024). Multi-institutional consensus on machine QA for isochronous cyclotron-based systems delivering ultra-high dose rate (FLASH) pencil beam scanning proton therapy in transmission mode. Med. Phys..

[pmbae023bbib56] Tan Y, Zhou S, Haefner J, Chen Q, Mazur T R, Darafsheh A, Zhang T (2023). Simulation study of a novel small animal FLASH irradiator (SAFI) with integrated inverse-geometry CT based on circularly distributed kV x-ray sources. Sci. Rep..

[pmbae023bbib57] Tang R, Yin J, Liu Y, Xue J (2024). FLASH radiotherapy: a new milestone in the field of cancer radiotherapy. Cancer Lett..

[pmbae023bbib58] Taylor P A, Moran J M, Jaffray D A, Buchsbaum J C (2022). A roadmap to clinical trials for FLASH. Med. Phys..

[pmbae023bbib59] Tessonnier T, Verona‐Rinati G, Rank L, Kranzer R, Mairani A, Marinelli M (2024). Diamond detectors for dose and instantaneous dose-rate measurements for ultra-high dose-rate scanned helium ion beams. Med. Phys..

[pmbae023bbib60] van Marlen P, Verbakel W, Slotman B J, Dahele M (2022). Single-fraction 34 Gy lung stereotactic body radiation therapy using proton transmission beams: FLASH-dose calculations and the influence of different dose-rate methods and dose/dose-rate thresholds. Adv. Radiat. Oncol..

[pmbae023bbib61] Vidal M (2024). Beam monitor chamber calibration of a synchro-cyclotron high dose rate per pulse pulsed scanned proton beam. Phys. Med. Biol..

[pmbae023bbib62] Vozenin M-C, Bourhis J, Durante M (2022). Towards clinical translation of FLASH radiotherapy. Nat. Rev. Clin. Oncol..

[pmbae023bbib63] Wei S, Lin H, Choi J I, Simone C B, Kang M (2021). A novel proton pencil beam scanning FLASH RT delivery method enables optimal OAR sparing and ultra-high dose rate delivery: a comprehensive dosimetry study for lung tumors. Cancers.

[pmbae023bbib64] Yan O, Wang S, Wang Q, Wang X (2024). FLASH radiotherapy: mechanisms of biological effects and the therapeutic potential in cancer. Biomolecules.

[pmbae023bbib65] Yang Y (2023). Commissioning a 250 MeV research beamline for proton FLASH radiotherapy preclinical experiments. Med. Phys..

[pmbae023bbib66] Yin L, Masumi U, Ota K, Sforza D M, Miles D, Rezaee M, Wong J W, Jia X, Li H (2024). Feasibility of synchrotron-based ultra-high dose rate (UHDR) proton irradiation with pencil beam scanning for FLASH research. Cancers.

[pmbae023bbib67] Zeng Y (2024). Feasibility study of multiple-energy Bragg peak proton FLASH on a superconducting gantry with large momentum acceptance. Med. Phys..

[pmbae023bbib68] Zhang Q (2023). Absence of tissue-sparing effects in partial proton FLASH irradiation in murine intestine. Cancers.

[pmbae023bbib69] Zou W (2021). Characterization of a high-resolution 2D transmission ion chamber for independent validation of proton pencil beam scanning of conventional and FLASH dose delivery. Med. Phys..

